# Reframing pandemic preparedness: an intelligence-led solidarity model for multi-layered emergencies

**DOI:** 10.1186/s40794-026-00310-6

**Published:** 2026-07-31

**Authors:** Alpha Umaru Bai-Sesay, Daniel Lavalie, Mohamed S. Bah, Patrick Maada Bundu, Aloubi Walter Emaka, Chizaram Anselm Onyeaghala

**Affiliations:** 1 Ministry of Health, National Public Health Agency, Freeown, Sierra Leone; 2 Research and Scientific Division, Sustainable Health Systems, Freetown, Sierra Leone; 3 Insights for Development Impact, Freetown, Sierra Leone; 4 Department of Internal Medicine, University of Port Harcourt Teaching Hospital, Port Harcourt, Nigeria

**Keywords:** Pandemic preparedness, Epidemic intelligence, Global health security, Health financing, Africa CDC, Multi-layered emergencies, Health systems resilience, Regional governance

## Abstract

The convergence of infectious disease outbreaks, climate shocks, and conflict is exposing fundamental limitations in existing global health preparedness systems, which remain largely fragmented and disease-specific. Epidemics increasingly occur within multi-layered emergencies, where overlapping risks interact to overwhelm fragile health systems and complicate response efforts. This perspective examines structural gaps in current preparedness approaches, including siloed financing, limited integration of multi-sectoral intelligence, and constrained institutional autonomy. Drawing on a structured policy analysis of publicly available literature and institutional experiences, particularly from the Africa Centres for Disease Control and Prevention (Africa CDC), the analysis highlights how integrated epidemic intelligence and regional coordination can support more anticipatory and context-responsive preparedness. Africa CDC’s evolving architecture illustrates the potential of combining surveillance data across health, environmental, and mobility domains to inform earlier and more targeted interventions. Building on these insights, this perspective proposes an intelligence-led solidarity model that integrates equitable resource allocation, interoperable regional intelligence, and predictable financing as interdependent components of resilient preparedness systems. Such an approach repositions preparedness as a shared global public good, emphasizing alignment between data, resources, and governance structures. In the context of increasingly complex and concurrent crises, an intelligence-led solidarity framework offers a pathway to strengthen global health security by enabling more coordinated, adaptive, and forward-looking responses.

## Introduction

The post-COVID-19 period has given way to overlapping crises that continue to test the limits of global preparedness. By March 2025, Uganda reported 4,881 confirmed mpox cases across 63 districts, while Sierra Leone reported over 4,800 confirmed cases [1–3]. Simultaneously, Eastern and Southern Africa faced one of the deadliest cholera epidemics in a decade, over 178,000 cases and nearly 2,900 deaths across 16 countries [4, 5]. Climate-driven floods, weakened sanitation systems, and population displacement have created conditions that facilitate disease transmission [6].

These are not isolated emergencies but interconnected phenomena. Epidemics increasingly unfold within multi-layered emergencies: crises where infectious disease, climate shocks, and conflict converge to overwhelm fragile systems. In South Sudan, for instance, recurrent flooding and protracted violence have displaced more than 1.8 million people, severely disrupting vaccine delivery and primary health care [7]. These conditions increase transmission risk while weakening already under-resourced health systems.

The global humanitarian architecture has struggled to respond. As of mid-2025, only about 14% of the US $47 billion required for the Global Humanitarian Overview had been funded [7, 8]. Financing mechanisms remain reactive, short-term and fragmented across vertical disease programmes. increased funding during crises followed by reduced investment in inter-epidemic periods [9]. The World Health Organization’s 2025–2028 General Programme of Work recognises epidemic intelligence and workforce strengthening as essential priorities [10]. However, these priorities require alignment of governance structures and financing mechanisms to be effective. Current approaches, still dominated by donor agendas and single-pathogen strategies, are limited in their ability to address the complexity of concurrent epidemics, climate emergencies and conflict-related displacement.

Current global health security architecture is largely structured around a vertical, pathogen-specific and donor-driven model. It is characterised by emergency response financing, short-term project cycles, and externally defined priorities. This model, rooted in post–Ebola and COVID-19 reforms, emphasises rapid containment of individual outbreaks through siloed programmes rather than sustained investment in system-wide resilience [10].

Although important mechanisms such as the International Health Regulations (2005) and the International Pathogen Surveillance Network have substantially strengthened global disease reporting, laboratory collaboration and pathogen sharing, they primarily establish legal obligations and technical surveillance functions. They do not by themselves provide integrated mechanisms that simultaneously align epidemic intelligence, predictable financing and equitable resource allocation across multiple institutions and regions. The intelligence-led solidarity model proposed here is therefore intended to complement, not replace existing global health architecture by providing an operational governance framework that links these currently disconnected functions.

While it has delivered important gains in outbreak control, growing evidence shows that it is poorly suited to the realities of multi-layered emergencies, where epidemics intersect with climate shocks, displacement and fragile governance systems [7, 9]. Importantly, vertical disease programmes have generated substantial public health gains, including improvements in laboratory capacity, surveillance systems, workforce development, and service delivery in many settings. The concern is therefore not the existence of vertical programmes themselves, but the limited coordination between these investments and broader preparedness systems during complex, multi-layered emergencies. Its structural limitations include fragmented surveillance systems, reactive financing mechanisms, and limited integration across sectors such as health, environment and humanitarian response.

The limitations discussed in this perspective arise from both structural design characteristics and implementation challenges. Existing international mechanisms have strengthened surveillance and reporting, but their implementation has often been constrained by political incentives, fragmented financing and inconsistent institutional coordination. The proposed model therefore focuses less on replacing existing institutions than on strengthening the operational linkages between governance, financing and intelligence that enable these mechanisms to function more effectively.

To address the complexity and simultaneity of modern crises. Drawing on the experience of the Africa Centres for Disease Control and Prevention (Africa CDC), this analysis advances an intelligence-led solidarity model rooted in regional autonomy, integrated data systems and equitable financing as a more adaptive approach to pandemic preparedness. Addressing these limitations requires a shift from fragmented response systems toward integrated, anticipatory preparedness approaches.

This perspective examines these gaps and proposes an intelligence-led solidarity model informed by emerging institutional experiences, particularly from Africa CDC, to better align epidemic intelligence, financing structures, and regional governance within a unified preparedness framework.

We apply a structured policy analysis approach to evaluate current preparedness limitations and develop an intelligence-led solidarity model grounded in empirical institutional experience.

Rather than proposing a new global institution, this perspective advances an operational governance framework designed to strengthen coordination among existing international, regional, and national preparedness mechanisms.

## Analytical approach

This perspective draws on a qualitative policy analysis approach to examine structural limitations in global health preparedness systems and to inform the development of an intelligence-led solidarity model.

The analysis is informed by the Walt and Gilson policy triangle framework, focusing on content, context, actors, and processes shaping global health security systems. This framework provides a structured lens to examine how governance arrangements, financing mechanisms, and institutional dynamics influence preparedness outcomes across settings.

This perspective draws on peer-reviewed literature, institutional reports (including those from the World Health Organization and Africa Centres for Disease Control and Prevention), and publicly available sources related to epidemic intelligence, health financing, and emergency preparedness. Sources were selected based on their relevance to global health security, epidemic intelligence systems, financing structures, and multi-sectoral emergency response, with a focus on evidence published between 2014 and 2025.

Evidence was thematically synthesised to identify recurring structural gaps in preparedness systems, including fragmentation in surveillance, financing constraints, and limitations in cross-sectoral coordination. These insights informed the development of an integrated conceptual model that links epidemic intelligence, equitable resource allocation, and predictable financing as interdependent components of resilient preparedness systems.

This approach enables structured evaluation of policy systems in the absence of primary data collection, while allowing for the integration of diverse evidence sources to support conceptual and policy-oriented analysis.

### Structural fragmentation in global health security

Despite repeated lessons from Ebola, COVID-19, mpox, and subsequent outbreaks, progress has been made in strengthening national and regional response capacities, particularly in countries such as Uganda. However, at the global level, emergency response architecture remains structurally fragmented, with financing, surveillance, and operational mechanisms often organised around disease-specific programmes rather than integrated risk management [7, 11].International funding continues to follow the logic of vertical, pathogen-specific programmes, with major financing streams such as outbreak-specific emergency funds and disease-targeted initiatives often operating in parallel rather than through integrated system-wide investments.

Inequities in resource allocation during public health emergencies remain well documented and are shaped by multiple structural factors. Global financing and access to countermeasures are often shaped by a combination of epidemiological risk, manufacturing capacity, regulatory readiness and geopolitical factors, rather than epidemiological burden alone [7].

During the 2022–2023 mpox outbreaks, high-income countries were able to rapidly access vaccines and therapeutics, partly due to existing procurement agreements and domestic manufacturing capacity. In contrast, countries where mpox is endemic, including the Democratic Republic of Congo (DRC), experienced more limited and delayed access, despite experiencing sustained transmission and higher case fatality in certain settings [4, 12]. While international support, including vaccine donations and diagnostics, was mobilised, responses were often reactive rather than anticipatory.

Large cholera outbreaks across multiple African countries have imposed substantial mortality and health system strain, yet global financing and media attention have varied across crises. Rather than reflecting a simple hierarchy of importance, these differences highlight structural challenges in aligning global response mechanisms with complex, overlapping risk profiles in multi-layered emergencies.

This reactive prioritisation can undermine preparedness in complex emergency settings. In flood-affected displacement camps, communities often face multiple concurrent risks, including cholera, measles, malaria, mpox and acute malnutrition. In practice, national governments, ministries of health and humanitarian coordination platforms, including humanitarian cluster coordination systems, work to align response efforts across these threats.

However, despite these coordination mechanisms, operational challenges persist. Donor-funded programmes frequently retain disease-specific reporting requirements, funding streams and performance indicators, which can limit integration at the point of service delivery. This can result in parallel implementation structures and missed opportunities for shared surveillance, joint logistics and integrated risk assessment. Strengthening interoperability across programmes, rather than replacing existing coordination systems, is therefore essential to improving efficiency and preparedness in multi-layered emergencies.

Dependency within global health financing structures further entrenches fragility. Crisis-specific funding surges when emergencies dominate headlines but quickly dissipates once global attention wanes, a dynamic long described as the panic-and-neglect cycle. This stop–start funding model prevents sustained investment in essential public health functions such as laboratory networks, genomic surveillance, and workforce training [11]. Consequently, subsequent outbreaks often occur in systems with limited improvements in core preparedness capacity.

Beyond inefficiency, the siloed approach obscures the reality that epidemics in Africa rarely occur in isolation. They interact with displacement, insecurity and ecological disruption to produce syndemics, situations where biological and social determinants amplify one another. Treating cholera, mpox, or malaria as discrete problems ignores the composite risk environment in which they unfold [4, 7, 10].

By failing to integrate epidemiological intelligence across sectors, the global health security system perpetuates inefficiency, inequity, and dependence. These limitations highlight the need for a transitionfrom vertical programming to multidimensional preparedness, anchored in data integration, shared accountability and regional ownership. Africa CDC’s experience demonstrates that such a shift is both feasible and increasingly important for strengthening preparedness in complex emergency settings in an era of cascading emergencies.

### Institutional case study – Africa CDC

In contrast to fragmented global responses, the Africa Centres for Disease Control and Prevention (Africa CDC) provides an institutional example of how regional coordination can support adaptive, intelligence-driven preparedness. Rather than constituting a standalone model, Africa CDC operates as a coordinating platform that works in partnership with national governments, ministries of health and international agencies to strengthen surveillance, preparedness and response capacities across the continent. Established in 2017 under the African Union, the agency was conceived specifically to strengthen regional capacity for surveillance, coordination and response in an environment where epidemics intersect with social and environmental shocks [10, 13].

At the core of this approach is the Public Health Intelligence Platform, a continent-wide system designed to consolidate data from national surveillance systems, climate monitoring, displacement tracking and health system indicators into a unified analytical interface. Unlike traditional disease bulletins that report cases in isolation, the platform enables correlation across data streams, for example, overlaying rainfall forecasts with cholera incidence, road accessibility and population-movement data to anticipate flare-ups before they spread [10, 13]. This multidimensional approach supports the translation of surveillance data into actionable insights.

During Uganda’s 2024–2025 mpox outbreak, national authorities, supported by Africa CDC, WHO and other partners, integrated surveillance data with mobility and environmental indicators to support identification of districts at elevated risk of transmission [14, 15].These insights supported national authorities and partners in pre-positioning diagnostics, vaccines and protective equipment. Available reports suggest that integrated data use contributed to more timely response coordination and improved resource targeting compared with earlier approaches [7, 15].

Africa CDC’s architecture also embodies decentralised regionalism. Five collaborating centres, North, West, Central, East and Southern Africa, adapt intelligence gathering to local contexts, languages and risk profiles. This proximity builds credibility and ownership among member states, attributes often lacking in externally directed initiatives. Regional hubs have begun integrating community-level reporting through mobile applications and leveraging local universities for field epidemiology training, thereby creating a continuous feedback loop between grassroots observation and continental analysis [10].

Beyond outbreak monitoring, Africa CDC, alongside national institutions and academic partners, is contributing to the growing use of predictive analytics that link climate variables to disease patterns and health system risks [7]. These developments illustrate how integrated intelligence approaches, implemented through collaboration between regional institutions, national systems and partners, can help bridge sectors such as health, environment and humanitarian response.

The agency’s evolution underscores a critical shift: preparedness extends beyond stockpiling vaccines or contingency planning to include anticipatory risk analysis and coordinated response systems. By converting fragmented information into anticipatory decision-making, Africa CDC demonstrates that intelligence is itself an essential public-health asset, one capable of supporting a transition from reactive response toward more resilient systems. This case illustrates how institutional architecture, when aligned with integrated data systems and regional governance, can operationalize anticipatory preparedness within complex emergency settings. While Africa CDC operates within the specific political and institutional context of the African Union, this perspective does not suggest that its institutional arrangements should be replicated identically elsewhere. Rather, the case illustrates a set of transferable governance principles that may be adapted to different regional contexts. These principles include integrating epidemic intelligence across sectors, strengthening regional coordination while respecting national sovereignty, promoting predictable institutional financing, and enabling collaborative decision-making across countries. Their implementation will necessarily differ according to regional political, legal, and institutional arrangements. The proposed intelligence-led solidarity model should therefore be understood as a flexible governance framework informed by Africa CDC’s experience rather than a prescriptive institutional blueprint for global adoption.

### Financing constraints and dependency

Despite its growing strategic relevance, the Africa Centres for Disease Control and Prevention (Africa CDC) operates within a financing environment characterized by substantial external support alongside structural funding constraints. Its annual budget, estimated at roughly US $150 million, remains substantially smaller than that of comparable institutionssuch as the US Centers for Disease Control and Prevention, which operate on more than US $12 billion annually. Yet the more profound challenge lies not only in scale but in the structure of financing [16]. A large proportion of Africa CDC’s resources are provided by external partners and are often earmarked for specific programmatic activities [17].

This financing pattern reflects a broader structural feature of global health systems. External funding has played a critical role in advancing public health outcomes across many low- and middle-income countries, supporting disease control, workforce development and emergency response. At the same time, the predominance of earmarked, project-based funding can present challenges for long-term system strengthening.

In particular, financing tied to specific outputs, such as commodities delivered or short-term programme targets, can limit flexibility for institutions to invest in cross-cutting functions including surveillance integration, workforce sustainability and infrastructure development. These dynamics are not unique to Africa CDC but are observed across multiple global and regional health institutions [11].

These constraints extend beyond administrative limitations. The structure of conditional and project-based financingcan influence priority-setting processes, sometimes favouring shorter-term, disease-specific outcomes over longer-term system investments.It may also shape the degree of operational flexibility available to regional institutions in aligning resources with context-specific priorities [7]. While Africa CDC’s legitimacy derives from regional ownership, its operational autonomy can be constrained by externally defined funding structures.

Comparable patterns are visible in other regional institutions, from the Caribbean Public Health Agency to the ASEAN Bio Diaspora Network, underscoring that dependency is a structural feature of the global health financing model, not an African anomaly. However, the implications are particularly acute for Africa CDC, given the continent’s disproportionate burden of emergencies and its pivotal role in continental health security.

Strengthening global health financing architecture to provide more predictable and flexible support alongside existing programmatic funding is essential to enable regional and national institutions to plan, adapt, and sustain preparedness efforts over time… These financing constraints highlight the need to align resource flows with integrated preparedness functions, reinforcing the case for a solidarity-based model that links financing, intelligence, and governance.

Importantly, the proposed model does not assume the availability of substantially new global resources. Rather, it advocates for improving the predictability, flexibility, and coordination of existing investments. A significant proportion of current global health financing is already directed toward preparedness, surveillance, disease control, humanitarian response, and health system strengthening through multiple funding streams. Greater alignment of these investments through pooled planning, multi-year financing commitments, and stronger coordination between global, regional, and national institutions could improve efficiency while reducing duplication and fragmentation. Additional financing may be required over time, particularly for low-resource settings, but the immediate priority is to optimize the use of existing resources while creating incentives for sustained domestic and international investment in preparedness.

### A solidarity-based model for resilient preparedness

Building on the preceding discussion, this perspective proposes an intelligence-led solidarity model that integrates three interrelated components: equitable resource allocation, integrated epidemic intelligence, and predictable financing. Rather than functioning independently, these components are mutually reinforcing and together support more anticipatory and resilient preparedness systems.

Breaking the entrenched cycle of fragmentation and dependency requires a shift in how health security is conceived, financed, and governed. A solidarity-based model seek to rebalance power, resources and intelligence, recognising that. Although pandemic preparedness is widely recognized within existing international frameworks as a shared global public good, translating this principle into sustained political commitment, predictable financing, and equitable implementation has remained challenging. The proposed intelligence-led solidarity model seeks to strengthen the operational mechanisms through which this shared commitment can be implemented. Building on Africa CDC’s experience, we propose a three-pillar structure that operationalises this model: equitable resource allocation, integrated regional intelligence, and predictable core funding.

#### Equitable resource allocation

The first pillar demands transparent and needs-driven distribution of vaccines, diagnostics, therapeutics and emergency financing. During the COVID-19 pandemic, Africa, home to 17% of the global population received only 8% of vaccine doses in 2021 [18]. A similar pattern resurfaced during the 2022–2023 mpox outbreaks, when high-income countries secured vaccine stocks through bilateral contracts while African countries facing higher mortality struggled to access supplies [19].

Within this model, resource allocation would be guided by *vulnerability indices* that integrate epidemiological data, climate risk and conflict exposure, rather than relying on market access or geopolitical leverage. Populations living in displacement camps, flood-prone zones or regions experiencing concurrent outbreaks should be prioritised for early intervention. Such a mechanism could be hosted under the auspices of WHO or a reformed global health financing body, but it should include mechanisms to discourage vaccine hoarding and export restrictions during crises and export restrictions during crises. Equitable allocation is both an ethical and strategic consideration, but also a strategic safeguard against the cross-border spread of preventable epidemics.

### Integrated regional intelligence

Preparedness is only as strong as the intelligence that informs it. adapting the governance principles demonstrated by Africa CDC and other regional public health networks through interoperable regional intelligence hubsin Asia, Latin America and the Middle East. These hubs would integrate health, climate, mobility and conflict data into predictive analytics capable of flagging high-risk convergence zones before outbreaks escalate.

Estimates suggest that an annual global investment of about US $500 million could establish and sustain such a network, a fraction of the trillions lost to pandemic disruptions. Importantly, this system would not rely on a monolithic global database but a federated architecture that respects data sovereignty while promoting insight sharing across regions [13]. Each hub would retain contextual control over its data while contributing to a collective situational picture of global health risk.

To ensure sustainability, such hubs must be embedded within regional institutions, such as ASEAN, PAHO or the African Union, rather than dependent on temporary donor projects. The resulting intelligence ecosystem would enhance global early warning, enabling pre-emptive resource deployment and faster decision-making across borders.

### Predictable core financing

The third pillar is financial predictability. Current funding mechanisms over emphasize emergency response while neglecting inter-crisis capacity building. Greater emphasis should be placed on flexible, multi-year core financing for regional and national preparedness institutions to ensure continuity of essential capacities between emergencies.

Such funding would allow countries to invest in enduring assets, laboratory networks, surveillance infrastructure, and workforce pipelines. Community health workers, for instance, have been shown to contribute to reduced mortality during outbreaks, yet they remain chronically underfunded because their contributions do not fit short-term project metrics [20]. Sustained core financing would ensure these frontline capacities are retained and trained between emergencies, transforming ad-hoc mobilization into institutionalized readiness.

Together, these three pillars represent more than a financial proposal they signal a shift in the moral and operational foundations of global health governance. In this model, solidarity is framed as mutual security: recognizing that resilience anywhere strengthens protection everywhere. In an age of converging crises, no nation can isolate itself from shared vulnerability. The transition from reactive emergency management to intelligence-led solidarity is therefore not aspirational, it is increasingly necessary. This framework provides a basis for aligning financing, data systems, and governance structures within a coherent preparedness approach suited to multi-layered emergencies.

### Operationalizing the intelligence-led solidarity model

Translating the proposed intelligence-led solidarity model into practice requires strengthening coordination across existing institutions rather than creating new global governance structures. The model is intended to complement established mechanisms, including the International Health Regulations (2005), the International Pathogen Surveillance Network, and regional public health institutions, by improving the alignment between epidemic intelligence, financing, and decision-making. Under this approach, the World Health Organization would continue to provide normative leadership and technical standards, while regional institutions such as Africa CDC, the European Centre for Disease Prevention and Control (ECDC), the Pan American Health Organization (PAHO), and relevant regional bodies in Asia and the Middle East would coordinate regional preparedness planning, intelligence synthesis, and cross-border operational support in collaboration with national governments.

Implementation would begin with the establishment or strengthening of interoperable regional intelligence hubs capable of integrating epidemiological surveillance with complementary information on climate hazards, population mobility, humanitarian emergencies, laboratory networks, and health system capacity. Rather than creating a centralized global database, these hubs would operate through a federated architecture that allows countries to retain ownership of their data while securely sharing standardized analyses and early-warning intelligence. This approach strengthens regional situational awareness while respecting national sovereignty and existing legal frameworks for data governance.

Financing should similarly build on existing global health financing mechanisms while addressing their current fragmentation. A blended financing approach would combine predictable multi-year institutional support for preparedness capacities with rapidly deployable contingency funding for emergencies. Flexible core financing would enable sustained investment in laboratory systems, workforce development, genomic surveillance, emergency operations centres, and integrated information systems during inter-epidemic periods, while emergency financing mechanisms would preserve the capacity for rapid response during acute crises. This combination reduces dependence on the recurrent cycle of emergency funding followed by periods of underinvestment.

Operational accountability would be embedded within existing monitoring mechanisms rather than creating parallel reporting systems. Countries and regional institutions could periodically report on preparedness investments, intelligence-sharing practices, and implementation progress through established monitoring frameworks, including the International Health Regulations Monitoring and Evaluation Framework, Joint External Evaluations, and other regional preparedness assessments. Monitoring these cross-cutting capacities alongside traditional preparedness indicators would help ensure that investments strengthen integrated system resilience rather than isolated disease programmes.

Lastly, continuous learning should be incorporated as a core feature of the model. Following outbreaks, simulation exercises, and public health emergencies, regional institutions and national authorities should jointly conduct after-action reviews to evaluate intelligence performance, financing effectiveness, and coordination across sectors. Lessons generated through these evaluations should be systematically integrated into preparedness planning, allowing governance arrangements, financing mechanisms, and intelligence systems to evolve in response to emerging risks. Through this iterative approach, the intelligence-led solidarity model functions not as a replacement for existing global health architecture but as an operational framework that strengthens the coordination, sustainability, and effectiveness of current preparedness systems.

The proposed implementation pathway is intended to be iterative rather than prescriptive, allowing countries and regional institutions to adapt its components according to their governance structures, resource availability, and public health priorities while maintaining the core principles of intelligence, solidarity, and accountability.

## Discussion

This perspective argues that strengthening pandemic preparedness requires moving beyond conceptual commitments to solidarity by embedding operational mechanisms that align governance, financing, and epidemic intelligence across existing institutions. Donor agencies, often operating within domestic accountability frameworks, tend to prioritize short-term projects with clearly measurable outputs. These dynamics can contribute to fragmentationeven when rhetoric supports equity.

Data sovereignty represents a significant consideration in the implementation of integrated intelligence systems. Governments may fear that sharing sensitive surveillance or mobility information may raise concerns related to security sensitivities and potential economic implications. Addressing these concerns requires transparent governance frameworks that guarantee national ownership of data while enabling its aggregation for public good. Federated architectures, where countries retain control but exchange anonymized intelligence, offer a feasible compromise already piloted by Africa CDC and the European CDC.

Another challenge is financial inertia. The global funding system remains reactive: crisis-driven appeals dominate while predictable contributions to core resilience remain exceptional. Yet the economic rationale for anticipatory investment is substantial. The COVID-19 pandemic alone cost the global economy an estimated US $12 trillion; establishing interoperable regional intelligence networks would require a small fraction of the economic losses associated with global pandemics of that amount annually.

Addressing these constraints will require both political commitment and institutional incentives that encourage sustained investment in preparedness. Multilateral development banks, philanthropic organizations, and bilateral partners could increasingly align financing with long-term preparedness benchmarks rather than short-term emergency outputs. Equally important, national governments should progressively increase domestic investment in core public health functions, consistent with their fiscal capacity, while regional institutions strengthen collective financing mechanisms among member states. Such shared responsibility distributes the financing burden across domestic, regional, and international actors, reducing dependence on any single funding source and improving long-term sustainability.

### Limitations

This perspective has several limitations. First, it is based on a qualitative policy analysis and synthesis of published literature rather than primary empirical data, and the proposed framework has not yet been prospectively evaluated in operational settings. Second, Africa CDC is presented as an illustrative institutional case study, and experiences from other regional organizations may differ according to political, legal, institutional, and financing contexts. Third, while the proposed intelligence-led solidarity model is intended to complement existing global health governance mechanisms, its implementation will inevitably require adaptation to regional priorities, institutional capacities, and political realities. Finally, the recommendations presented are conceptual and policy-oriented and should therefore be interpreted as a framework to stimulate discussion and future implementation research rather than a universally applicable governance blueprint. Future empirical studies should evaluate the feasibility, effectiveness, cost implications, and implementation outcomes of intelligence-led solidarity approaches across diverse regional and national settings. Ultimately, the success of the proposed framework will depend not only on its technical design but also on sustained political commitment, mutual accountability, and long-term investment by national governments, regional institutions, and international partners.

## Conclusion

The converging forces of epidemics, conflict, and climate instability have highlighted the limitations of fragmented global health responses. Traditional, disease-specific systems designed for single hazards are not well suited to address the complexity of multi-layered emergencies now confronting the world. The experience of the Africa Centres for Disease Control and Prevention demonstrates how regional institutions can operationalize integrated preparedness within their respective political and institutional contexts. Although implementation will necessarily vary across regions, the underlying principles of coordinated intelligence, equitable resource allocation, and predictable financing have broader relevance for strengthening global preparedness.

However, the potential of such institutions remains constrained by structural features of global health governance and financing. Current systems often prioritize short-term outputs and emergency visibility, while underinvesting in the foundational capacities required to sustain resilience between crises. Without more predictable and flexible support, regional institutions risk operating with limited strategic autonomy.

Strengthening global health preparedness will require more effective operationalization of the widely accepted principle of global solidarity through stronger governance, sustainable financing, integrated intelligence, and greater institutional accountability. Equitable allocation, interoperable intelligence, and predictable core financing, together, constitute key components of a more coherent preparedness architecture. Supporting regional resilience through integrated intelligence, equitable financing, and coordinated action represents a practical pathway for strengthening global preparedness.

Future empirical research should evaluate the feasibility, effectiveness, cost implications, and equity impacts of intelligence-led solidarity approaches across diverse regional and national contexts to determine whether they strengthen preparedness beyond existing governance arrangements.

## Data Availability

No new data was generated or analysed in this analysis. All information referenced in this manuscript is derived from publicly available sources, which are appropriately cited in the reference list.
